# Short-Term Psycho-Education for Caregivers to Reduce Overmedication of People with Intellectual Disabilities (SPECTROM): Development and Field Testing

**DOI:** 10.3390/ijerph182413161

**Published:** 2021-12-14

**Authors:** Shoumitro (Shoumi) Deb, Bharati Limbu, Gemma Unwin, Linda Woodcock, Vivien Cooper, Michael Fullerton

**Affiliations:** 1 Department of Brain Sciences, Faculty of Medicine, Imperial College London, Du Cane Road, London W12 0NN, UK; b.limbu@imperial.ac.uk; 2 School of Psychology, University of Birmingham, Edgbaston, Birmingham B15 2TT, UK; g.l.unwin@bham.ac.uk; 3 AT-Autism, 20-22 Wenlock Rd, Hoxton, London N1 7GU, UK; lindawoodcock@atautism.org; 4 The Challenging Behaviour Foundation, The Old Courthouse, New Rd Ave, Chatham ME4 6BE, UK; vivien.cooper@thecbf.org.uk; 5 Achieve Together, Q4, First Floor, The Square, Randalls Way, Leatherhead KT22 7TW, UK; michael.fullerton@achievetogether.co.uk

**Keywords:** intellectual (learning) disabilities, adults, support staff, training, psychotropic medication, alternatives to medication

## Abstract

People with intellectual disabilities (PwID) are at a higher risk of developing challenging behaviours (CB). Despite the poor evidence for the effectiveness of medications in managing CB, they are used widely among PwID (50–63%). The aims of our study were to develop a training programme, SPECTROM for support staff to help reduce overmedication in PwID and carry out field testing of SPECTROM including a process evaluation. We developed SPECTROM using the Experience-based co-design method that included four focus groups and a one-day co-design event. Twenty trainees received SPECTROM training. We used the Management of Aggression and Violence Attitudes Scale-Revised-Intellectual Disabilities (MAVAS-R-ID) and the Psychotropic knowledge questionnaire. A semi-structured interview and a feasibility questionnaire were used for process evaluation. SPECTROM website contains 14 modules, resources, and face-to-face training. MAVAS-R-ID scores showed change in staff attitude to ‘medication management’ domain was statistically significant (*p* < 0.05). Psychotropic knowledge questionnaire showed statistically significant post-training improvement in correct responses (*p* < 0.05). Process evaluation data showed that SPECTROM was acceptable, applicable, practical, and relevant to staff practice, and helped to improve self-reflection, knowledge, and support to PwID. SPECTROM is a useful training that helps to change the support staff’s attitude toward CB and improve their knowledge of psychotropic medications.

## 1. Introduction

Individuals with intellectual disabilities are at a higher risk of developing behaviours that challenge (challenging behaviours) (18–22%) [1,2] including aggression to others, property, and self [3]. Challenging behaviour poses a major management problem and is an obstacle to social integration, and it may lead to carer stress, community placement breakdown and hospitalisation, and the use of restrictive practices such as physical restraint and inappropriate use of medication. To address challenging behaviours, it is important to understand the reason behind them rather than use medication to sedate. A thorough person-centred assessment with multidisciplinary input is vital for a formulation leading to an appropriate person-centred support package that helps to improve the behaviour and the quality of life of the person with intellectual disabilities [4]. Among other factors, poor staff training, and organisational policies are shown to be the important factors in the successful withdrawal of psychotropic medications [5]. Proper training and support for staff are thus of paramount importance for a successful programme of rationalisation of psychotropic medication use in adults with intellectual disabilities.

Both pharmacological [6] and psychosocial interventions such as Positive Behaviour Support (PBS) [7] are used to manage challenging behaviour. A recent meta-analysis found a significant long-lasting moderate overall effect of non-pharmacological interventions such as mindfulness, behaviour techniques, and PBS on challenging behaviours (effect size = 0.573) [8]. In contrast, the evidence for the effectiveness of medication in managing challenging behaviour is at best equivocal [6]. Nevertheless, psychotropic medications are used widely among people with intellectual disabilities (50–63%), often off-licence [9]. However, UK national [10] and international [11] guidelines on the management of challenging behaviour among people with intellectual disabilities recommend the use of medication only when psychosocial interventions have failed and there are serious risks of harm to self or others.

The off-licence use of antipsychotic medications in people with intellectual disabilities is a major public health concern worldwide [11]. In the UK, it has been estimated that every day around 35,000 adults with intellectual disabilities receive psychotropic medications unnecessarily [12]. Because of these concerns in the UK, the National Health Service England has embarked on a major campaign called ‘STopping Over-Medication of People with ID, autism, or both (STOMP)’ [13]. Appropriate staff training is successful in reducing psychotropic medication use in people with dementia [14] and improve autism symptoms in children through parental training [15]. The current training available to support staff working with adults with intellectual disabilities including PBS training do not directly address the issue of overmedication [16].

To address the aforementioned issues, we have recently developed “Short-term Psycho-Education for Carers To Reduce Over Medication of people with intellectual disabilities” (SPECTROM) training programme using a co-production method to help support (care) staff in community homes to facilitate the reduction in the overmedication of psychotropic medication among adults with intellectual disabilities [17].

## 2. Materials and Methods

The main objectives of our study were to: (a) develop a training programme, SPECTROM according to Medical Research Council’s (MRC’s), UK [18] guideline for the development and evaluation of the complex intervention; (b) carry out a field testing of SPECTROM; and (c) conduct a process evaluation to gather feedback from the participants on the training to assess implementation issues. In this paper, we will present, primarily, data on field testing and the process evaluation. Further details of the methods used to develop SPECTROM are available in Deb and colleagues’ paper [17] and a summary is presented below.

### 2.1. Development of SPECTROM

SPECTROM was developed using a modified Evidence based co-design (EBCD) methodology [19] involving focus groups, co-design event, and synthesis of existing evidence. All the relevant stakeholders were involved in the study, which included (a) adults with intellectual disabilities and their families; (b) support staff working with adults with intellectual disabilities in community settings and their service managers; (c) Community Learning Disability Team (CLDT) members; (d) general practitioners; (e) pharmacists; and (f) six social care provider organisations; the Chief Executive of a family caregiver organisation and a Director of an organisation, AT-autism.

We collated information on the existing training programmes to avoid unnecessary duplication. Two groups took part in focus groups that were held in London, UK. Group 1 comprised support staff only (*n* = 8); and Group 2, service managers and trainers (*n* = 8). Each group attended two sets of focus groups. The first set explored the attendee’s experiences and perception of the use of medication to manage challenging behaviour in people with intellectual disabilities, and the second set concentrated on the attendee’s suggestions regarding the contents and the format of SPECTROM. Following the analysis of focus group data using a thematic approach [20], a one-day co-design event was held in London, UK to clarify several themes that were generated from the focus groups. The co-design day included five groups of six stakeholders in each (support staff, family carers, trainers, consultant psychiatrists, CLDT members, and service managers). Feedback from people with intellectual disabilities was received through the Cornwall Learning Disability Advisory Group (LDAG).

Data from the review of existing training, focus groups and co-design event day were analysed and collated by the core research team. The SPCTROM development group, consisting of 14 stakeholders, synthesised this information and made recommendations to the core research group for the development of draft SPECTROM modules. The draft was sent to 59 stakeholders via email for their comments which were incorporated into the final version of SPECTROM.

### 2.2. Field Testing

Four trainers provided face-to-face training to a total of 20 support staff in groups of 2–7 participants. Support staff from the UK National Autistic Society in Scotland (NAS) (*n* = 3), AT-autism (*n* = 2), and Challenging behaviour foundation (CBF) (*n* = 7) participated in the training. Additionally, support staff from the various service provider organisations in Australia took part in the training, facilitated by the Australian NDIS Quality and Safeguards Commission (*n* = 8).

Outcome data were collected using two questionnaires before delivering the training and within a week of post-training. An adapted ‘Psychotropic knowledge questionnaire’ [21] (Supplementary Material S1) was used to assess the knowledge of psychotropic medication use in adults with intellectual disabilities. The ‘Management of Aggression and Violence Attitude Scale-Revised-Intellectual Disabilities (MAVAS-R-ID)’ (Supplementary Material S2) that was adapted from ‘MAVAS-R’ [22] was used to assess participants’ change in attitude toward challenging behaviour and the person behind the behaviour. 

Psychotropic knowledge questionnaire has ten questions on medication and six on adverse effects and MAVAS-R-ID has 17 questions altogether. Psychotropic knowledge questionnaire items were scored as either a right answer (=1), or a wrong answer (=0), or a ‘don’t know’ answer (=0). MAVAS-R-ID was scored using a 5-point Likert scale (‘strongly agree’ to ‘strongly disagree’). MAVAS-R-ID items are divided into five domains; (1) internal causative factors, (2) external causative factors, (3) situational/interactional causative factors, (4) management-medication, and (5) management-non-medical. We have combined the scores on ‘agree’ and ‘strongly agree’ under a composite score on ‘agree’, and ‘disagree’ and ‘strongly disagree’ scores in a combined ‘disagree’ score. A higher score or rate of ‘disagreement’ indicates a better outcome for domains 1 and 4 and the opposite is the case for domains 2, 3, and 5. The maximum possible score on the psychotropic knowledge questionnaire is 16 and on MAVAS-R-ID, 85.

A purpose-designed questionnaire (Supplementary Material S3) was designed to capture trainees’ views on four domains, applicability, acceptability, practicality, and relevance of SPECTROM. A free-text box was also provided for the participants to write comments. Each question was scored on a 5-point Likert scale (‘Disagree completely’ = 1, ‘Disagree somewhat’ = 2, ‘Neither agree nor disagree’ = 3, ‘Agree somewhat’ = 4, and ‘Agree completely’ = 5). A total maximum score of 190 is possible and a combined ‘Agree somewhat’ and ‘Agree completely’ score is above 114 which is considered to be a good response. As for the domains (a) a total maximum score of 85 in the ‘applicability’ domain is possible and any score over 51 (a combined ‘agree’ score) is considered as a good response; (b) a total maximum score of 50 in the ‘acceptability’ domain is possible and any score over 30 (a combined ‘agree’ score) is considered as a good response; (c) a total maximum score of 40 in ‘practicality’ domain is possible and any score over 24 (a combined ‘agree’ score) is considered as a good response; and (d) a total maximum score of 15 in relevance’ domain is possible and any score over 9 (a combined ‘agree score) is considered as a good response.

A purpose-designed questionnaire (Supplementary Material S4) similar to the one used for trainees was developed for collecting feedback from trainers using the same domains and a similar scoring system, but some questions in this scale differed from that in the trainee’s questionnaire. Additionally, a purpose-designed proforma (Supplementary Material S5) was used to capture trainers’ pre-training (e.g., the time needed to prepare for presentation) and during training (e.g., the time needed to deliver the training, contents, and format of the training material and the manuals) experience.

The researcher (BL) also carried out interviews with five trainees using a topic guide (Supplementary Material S6). The interviews were transcribed, and qualitative data were analysed using a thematic analysis method [20]. The NVivo 12 plus software (QSR International, Melbourne, Australia) was used to help with the data coding.

## 3. Results

### 3.1. SPECTROM

SPECTROM web-based materials consist of (a) modules and (b) internal and external resources. Two core modules are (a) Medication, and (b) Alternatives to medication (ATM) (https://spectrom.wixsite.com/project, accessed on 1 August 2021). Other modules include: (1) Medicine review/STOMP action plan, (2) Medicine withdrawal review, (3) Assessment of behaviour and the person behind the behaviour, (4) Effective liaison with family carers and advocates, (5) Effective liaison with professionals (GP, Community team members and psychiatrists), (6) ATM-Introduction (Functional analysis, Positive Behaviour Support-PBS, etc.), (7) Communication needs of the person with intellectual disabilities, (8) Effective engagement with and support for the person with intellectual disabilities, (9) Psychiatric disorders vs. challenging behaviour, (10) Physical disorders vs. challenging behaviour, (11) Autism Spectrum Disorder (ASD), and (12) Attention Deficit Hyperactivity Disorder (ADHD). SPECTROM also include many hyperlinked external resources and internal resources such as accessible psychotropic medication leaflets, Comprehensive Assessment of Triggers for behaviour of concern Scale (CATS) [23], and the Yellow Book, which is a patient/carer handheld health passport containing all relevant health related information in an accessible format that can be taken to a doctor’s clinics and hospital appointments. The core modules are delivered face-to-face/virtually through which the other modules are introduced. Unlike traditional training, SPECTROM is designed not for a one-off training session but for long-term learning through frequent reference to the materials during day-to-day person-centred care planning for adults with intellectual disabilities. 

Both the web-based modules and core training modules consist of didactic PowerPoint presentations, case vignettes, group discussions, group and individual activities, video clips, reference to external resources, and Multiple-Choice Questions (MCQs). Core modules accompany very detailed manuals and PowerPoint slides so that the training could be provided either online using the SPECTROM site directly or offline using a computer. Each core module also provides several handouts for the trainees and summary points/take-home messages at the end. The trainees are encouraged to explore the materials on the SPECTROM site in more detail as part of their homework. Our field testing has shown that each core module can be presented in one day.

### 3.2. Field Testing

Staff from NAS completed only the ATM core module, AT-autism completed only the Medication core module. Both the CBF (*n* = 7) and the Australian trainees (*n* = 8) completed both core modules. In total, 18 participants completed the ATM core training and 14 Medication core training. There were six direct support staff and two service managers/team leaders. The rest included family support staff, the Chief Executive of CBF, and one advocate.

### 3.3. Psychotropic Knowledge Questionnaire

The Psychotropic knowledge questionnaire was completed by 13 participants. The pre-training scores ranged from 2 to 12 and had one missing response. The post-training scores ranged from 8 to 14 and had one missing response in 7 returned questionnaires. The missing answers were not included in the overall score.

**Figure 1:**
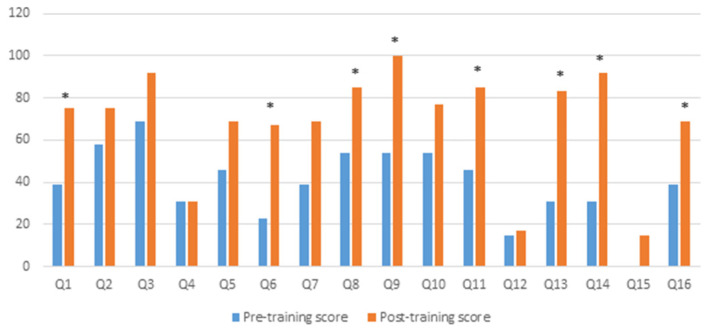
% correct responses on psychotropic knowledge questionnaire pre and post training. * *p* < 0.05.

As data were not normally distributed, the Wilcoxon signed-rank test was used to analyse data that showed participants scored significantly more correct answers after the training (*Median* = 10; *IQR* = 9–12.50) than before the training (*Median* = 6; *IQR* = 3–8.50), (*Z* = −2.753, *p* = 0.006). Figure 1 shows the percentage of correct answers for each question before and after the training. After the training, the percentage of a correct answer increased for each question, except for question 4 (How much time do antipsychotics generally take to show a positive effect on the psychotic symptoms of patients?), which remained the same (31%) before and after the training. The highest percentage (100%) of the correct answer was found for question 9 (Some antiepileptic drugs are also used for treating challenging behaviour). In eight out of 16 questions (50%), the post-training improvement in correct answer scores were statistically significant (*p* < 0.05) (see Figure 1).

### 3.4. MAVAS-R-ID

**Figure 2:**
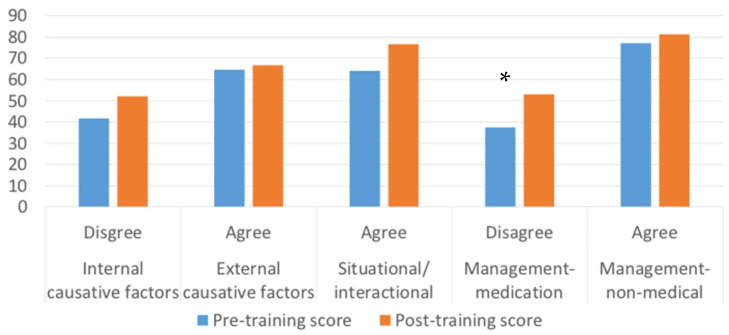
Number of only ‘best responses’ pre and post training in % for each domain in MAVAS-R-ID. A higher ‘disagree’ score for internal causative factor and management-medication, and a higher ‘agree’ score for external, situational/interactional causative factors, and management non-medical mean an improvement in staff attitude towards challenging behaviour and the person with intellectual disabilities. * *p* < 0.05.

MAVAS-R-ID was completed by 16 participants. Wilcoxon signed-rank test was used to assess post-training score change as data were not normally distributed (see Table 1). Only one domain score (medication-based management) showed a statistically significant better result at post-training (*Median* = 14.50; *IQR* = 12–16) when compared with the pre-training score (*Median* = 13; *IQR* = 11–15.75), (*Z* = −2.039, *p* < 0.05). However, domains 1 and 4 showed a higher post-training combined ‘disagree’ (‘Disagree completely’ + ‘Disagree somewhat’) responses whereas domains 2, 3, and 5 showed a higher combined ‘agree’ (Agree somewhat’ + ‘Agree completely’) responses after training. Although only one of these reached a level of statistical significance, they all show improvement in attitude after the training (see Figure 2). The Figure 2 shows there were more ‘best responses’ being made post-training, which suggest an improvement in staff attitude after the training.

### 3.5. Trainee Questionnaire Data

The trainee questionnaires were completed by 12 participants. All participants scored above the neutral value of 114 (neither agree nor disagree), meaning a score on either ‘agree somewhat’ or ‘agree completely’, which indicates a good outcome. The mean and standard deviation (SD) of the total score and proportion showing over the neutral value are presented in Table 2. Cronbach’s alpha of total and domain-based items was tested for internal consistency which showed satisfactory to excellent values for all items (see Table 2). 

#### Free Text Box

Four participants made comments in the free text section. One comment related to lack of time to go through all the resources available in SPECTROM as there is a lot to pursue. One mentioned that frequent breaks during the face-to-face training session helped her to concentrate on the amount of material presented. Two others mentioned that they found the training interesting and ‘the website easy to navigate through with resources readily available for learners to find more information’.

### 3.6. Trainer Questionnaire Data

Three out of four trainers (75%) completed the trainer questionnaire. All scored above 126 according to the Likert scale scoring either ‘somewhat agree’ or ‘agree completely’ (see Table 3). Cronbach’s alpha was not calculated due to the lack of trainers’ number.

#### 3.6.1. Applicability

All trainers agreed that the training will be useful for support staff’s day-to-day practice, give them the confidence to ask doctors the right questions, help them understand the side effects of psychotropic medication better, understand the person they support better and the reasons for challenging behaviour better. All trainees also agreed that the accessible psychotropic medication leaflets are useful in explaining medications to people with intellectual disabilities and that external resources are useful for gathering important information that could be used in support staff’s day-to-day practice. All trainees agreed that the training will help to change support staff’s practice for better and improve their attitude to challenging behaviour, and the person manifesting the behaviour.

#### 3.6.2. Acceptability

The trainers agreed that it was easy to prepare for the SPECTROM training. They found the core modules were easy to understand and the contents were appropriate and useful, particularly the handouts, group discussions, trainee activities, video clips, and case studies. One trainer stated that it took more time to complete the training than anticipated.

#### 3.6.3. Practicality

All trainers agreed that the information in SPECTROM will help support staff to gain confidence in carrying out the staff team’s in-house medication review regularly and the information can be used as reference points when discussing person-centred care planning for adults with intellectual disabilities. They also agreed that the training will help support staff to liaise better with relevant professionals and family caregivers. They also agreed that the training will help support staff engage better with the person they support and concentrate on their skills building rather than the challenging behaviour.

#### 3.6.4. Relevance

All trainees agreed that they would recommend the training to others, and the training complements other existing training in the service. They also agreed that the training is relevant to support staff’s personal development and should help eventually to reduce overmedication of people with intellectual disabilities.

#### 3.6.5. Free Text

Only one trainer included further information in the free text box. The trainer said, “The training is easy to follow, and the resources are excellent. Staff found the booklet to prepare for a doctor’s appointment most useful. They thought it would be worthwhile having videos for the Australian context rather than the UK as there are subtle but important differences.”

### 3.7. Trainer’s Proforma Data

As the trainer’s proforma questions were not scored numerically, no quantitative data could be presented but instead, we have summarised their overall response to the questions in the proforma. The time taken to prepare for the training varied between 1 and 5 h and 30 min and 3 h to read the corresponding training manuals. All trainers found that the manuals were easy to follow and had clear information on how to deliver the training, and the contents of the core modules were comprehensive and useful. However, it was noted by one trainer that the trainer needs a good base knowledge of psychotropic medications and PBS to deliver the medication core module. The format of the training helped to engage participants and led to discussion, which the participants found useful. Two trainers completed the training in 6 h respectively and one trainer completed the training in less than four hours. One trainer found the handouts lengthy, while the other two trainers found the handouts useful. However, all trainers agreed that the participants found the handouts useful to keep as a reference point.

All trainers found the trainee activities and case studies useful in encouraging group discussions. As for the video clips, one trainer found them useful, particularly in giving trainees a break from didactic learning. One trainer could not show the videos due to technical issues and the Australian trainer felt that the videos could be adapted using the Australian context. All trainers stated that they were able to engage the trainees adequately in the discussion. Two trainers did not skip any content while one skipped the trainee activity on self-care. All trainers found the contents of the modules are at the right level of support staff’s knowledge and expectation and the sessions were well-paced, so they did not have to rush the training.

In terms of challenges faced during the training, one trainer had only two trainees. Therefore, although the discussions were limited, they were more in-depth due to the small group. One trainer felt that there were a lot of materials to go through which could potentially limit discussions, but the trainer found that the case studies were situated at the right places to balance between the didactic learning and trainee engagement. All trainers stated that they and their trainees found the accessible medication leaflets useful in explaining medication-related information to people with intellectual disabilities. All trainers and their trainees found the Yellow Book useful, while one thought it was UK specific and requires amendment. One trainee commented during the training session, “Yellow Book is useful in storing all information in one place and all services should have access to this and take it with them to hospitals or in places where the professionals do not know the person with intellectual disabilities well.” All trainers found the external hyperlinked resources were useful, while one thought some of them were UK-specific.

Two trainers wrote comments in the free text box. One liked the training and felt that the training encouraged self-reflection as the trainer said, “What I liked most was having quality time with staff and the group discussions which led to some really useful self-reflection on both my and the staff’s part. There was nothing I didn’t like about the training.” The other trainer liked the format of the training and resources as the trainer said, “The training is well thought out and easy to follow and the resources are excellent.” The same trainer found “Alternatives to medication session is a bit slow” but mentioned, “the trainees liked it”.

### 3.8. Interview Data

Five trainees took part in the process evaluation interviews, four of whom were female and one male. All interviewees felt that SPECTROM training was useful. The main themes for the impact of training identified are presented here with some quotes. Interviewees also felt that the resources available on the SPECTROM website were useful and relevant to them.

#### 3.8.1. Changes to the Attitude in Addressing Challenging Behaviour

Participants mentioned that the training helped to “look at challenging behaviour in a different way and put things into perspective”, “provided insight to challenging behaviour and ways to address this”. One participant said, “It’s a new approach in looking at things and how we can manage it the way we can before it reaches crisis point…. It was an eye opener to learn about things that would benefit the young adults and benefit the staff as well.” “It opens your eyes on what can potentially be making them upset that we’ve never thought about.”

Participants mentioned that the training helped to encourage non-medication approaches such as PBS for supporting the person when they are distressed. Trainees felt that the training should help to reduce restrictive practices, overmedication of people with intellectual disabilities, and help them to understand the risks involved in using medication better. One participant said, “I feel guilty about giving medications… without even considering how it would affect the person.”

Interviewees also mentioned that the training helps to “analyse and explore triggers that may impact challenging behaviour”. Thus, helps them to address challenging behaviour more effectively. One interviewee said, “It helps us see things that we’ve never thought about, as simple as it may seem… some things that the young people could cope with, which we never thought.”

#### 3.8.2. Improvement in Self-Reflection

There was a consensus that the training helped support staff to reflect on their current practice as to how they address challenging behaviours in people they support, their own behaviours and stress, and the support they were providing to people with intellectual disabilities, particularly concerning their attitude toward the challenging behaviour and the person who manifests the behaviour. Interviewees said that previously they “did not consider the effects of medications” on people they support. They reflected on their past behaviour, where they have used medications for challenging behaviour but now felt empowered to question whether medications are still necessary. One interviewee said, “Until, it’s pointed out to you, you don’t realise what you were doing…” Another said, “If I’m not in a right mind, whatever it is, it will have an impact on the clients. It is good to reflect on yourself, just not the client. The focus is often on the clients. We as carers might come home stressed and it will have an impact. It will translate immediately to the client… the impact is on the client behaviour if the staff is not well aware of themselves”.

#### 3.8.3. Improved Knowledge

The interviewees felt that the training was informative and helped them improve their knowledge of different psychotropic medications and their side effects. However, it was just not the medication, they also learned about the medication review process, triggers for challenging behaviour, and caregiver-related factors that can affect the behaviour of the person they support. One participant said, “I was eager to learn my job and I learned my job with this training… they come together and form a pass for me to master what I am doing”.

#### 3.8.4. Improve the Support Provided to People with Intellectual Disabilities

Interviewees mentioned that the training improved their understanding of the person they support that led to a better person-centred approach for addressing challenging behaviour. The training also helped the interviewees to improve the support they provide to people with intellectual disabilities in a way that encourages people with intellectual disabilities to be more skilled and independent, explore and overcome triggers for challenging behaviour, and help with the person’s community integration. “We only moved here half a year ago… with a young man to integrate back to the community, our work aspects and responsibility has changed because we are in the community setting. I think that training would’ve been great before we moved because it would be very beneficial in promoting independence and behaviour wise… but now moving to adult services, it will be great training for them, because you need to become more person centred and know how to overcome triggers and behaviours”.

#### 3.8.5. Empowerment and Improved Confidence

The interviewees felt empowered to advocate on behalf of the person they support as they were informed on the impact and consequences of medications. They felt as if they had achieved a “voice” through this training and the information in the training gave them the confidence to ask psychiatrists or other professionals any necessary questions. One participant said, “I’ve been doing this kind of work close to 10 years now and I wish I had this training a long time ago…. I wished I’d known the information about medication, so I could’ve said something…” Another said, “I can now use my knowledge to ask questions if I go to a doctor appointment, I can ask right questions and somebody there who is doing the review will understand. If I go to a panel review, I will ask why is that client given that medication, what have you done before that, what are the strategies, and did you try any other alternatives, I can ask those questions”.

#### 3.8.6. The Overall Experience of Receiving SPECTROM Training

Although the interviewees felt that the training was informative and relevant to the trainees’ roles, 60% (3/5) participants said the training was at the right level for them and all terminologies were explained; and 40% (2/5) felt the contents were below their level of experience, but the training helped them to reflect on their practices. One participant said that the information from the training could also be used in future career roles. The interviewees found the training relaxing in nature and interactive as they enjoyed the back-and-forth discussion format of the training. They felt that the training was well-structured, and the bullet points helped to simplify the information. The comfortable atmosphere and small group size helped to facilitate discussions in the training and allowed input of different viewpoints.

Resources available on the SPECTROM website were seen overall as useful by all interviewees. They found that the accessible medication leaflets were “easy to understand”, “useful at gathering information on medication”, and “easy to carry around”. Case studies helped participants to reflect on what was learned and helped to test their knowledge. Handouts helped to recap and refer back to the training. Interviewees found it was practical to have all the information including medications “all in one place”, which could be done by printing hard copies and keeping them in one folder within the service. They found the SPECTROM website useful, as it includes many useful resources, and is also easy to navigate through. They also liked the fact that they can access these resources anytime they want, which will clarify issues during person-centred care planning or staff-led in-house medication reviews.

However, due to the COVID-19 pandemic, which increased staff’s workload, interviewees found it difficult to complete the homework and view all the resources available on the SPECTROM webpage. Hence, many could not view the resources on the website because of the lack of time. Other factors included lack of computer at workplace and internet connection issues due to the location of the services. One interviewee said that the managers need to be involved to implement the use of SPECTROM resources by support staff in the service. The main issue was that staff did not have time at work or at home to access further resources and, hence, needed managerial and organisational support to access and implement these resources. As stated at the outset, it is important to emphasise that SPECTROM is not designed for a one-off training session but should be used as an ongoing source of information while providing person-centred care for people with intellectual disabilities. Interviewees felt that SPECTROM complimented the approach used and training available in the services, such as PBS, and are in line with many services’ core ethos. Thus, all interviewees stated that they would recommend the training to their colleagues.

## 4. Discussion

Our aim was to develop a training programme for support staff in community homes to help to reduce the overuse of psychotropic medication among adults with intellectual disabilities and carry out field testing in preparation for a future definitive randomised controlled trial (RCT). This was achieved by putting stakeholders’ experiences at the centre of the study and ensuring close and equal collaboration among them. The ultimate aim was to empower, inform, and equip support staff with skills to understand challenging behaviour, its causes and the person manifesting the behaviour, manage their own psychological responses to behaviour and negotiate the care pathway, advocating on behalf of the person for whom they care, and taking the views of adults with intellectual disabilities fully into account.

The two main objectives we achieved with SPECTROM were educating support staff on alternatives to medication such as PBS [7] to address challenging behaviour, and equipping them with the skills to conduct in-house staff team medication reviews regularly. These will help staff to go fully prepared for the formal medication reviews carried out by doctors, and enable them to provide much better information. It is not uncommon now for staff to attend clinics with very little knowledge of the person they accompany. Well-informed medical reviews should help to reduce inappropriate psychotropic prescribing. Other important aspects of SPECTROM are: to improve staff engagement with the person with intellectual disabilities, their families, and other professionals; to understand the person behind the behaviour rather than concentrate on the challenging behaviour itself; to and engage in the development of skills including communication and social interactions with the person they support.

Although several small studies of staff training have shown some impact on challenging behaviour [16], SPECTROM is the only programme specifically developed to help staff to play a direct part in facilitating the reduction in inappropriate prescribing. In a previous review of staff training in intellectual disabilities, Deb and Roberts [24] found that whereas most training increases staff knowledge, it does not necessarily help to change staff attitudes towards challenging behaviour. SPECTROM is designed not only to improve staff knowledge but more importantly to change staff attitudes toward the use of medication to address challenging behaviour. Although SPECTROM is based on PBS [7] and person-centred principles, the training goes way beyond traditional PBS training [25], and it provides many more resources than the standard PBS training. The PBS framework [26] is very much focussed on developing a PBS approach with service users. It does not do all the things that SPECTROM does. The framework also acknowledges that PBS has not been implemented faithfully. SPECTROM will, in turn, provide the necessary platform to properly implement PBS. SPECTROM also teaches staff how to recognise their own stress in reacting to challenging behaviour and how to address it. This in turn breaks the negative cycle of challenging behaviour and teaches staff to take a non-confrontational approach to challenging behaviour, thus initiating a positive cycle.

In our study, various aspects of support staff’s beliefs were explored to influence beliefs to encourage positive behaviour, which was measured by assessing staff’s beliefs, particularly controllability of, and attribution to, the challenging behaviour. Therefore, our aim was not only to provide the trainees with the necessary knowledge about medications and the alternatives to medication, but also to change their attitude towards the person who displays challenging behaviour.

The field-testing questionnaire assessment showed an increase in psychotropic medication use knowledge after the training, which is expected. The proportion of correct answers increased for all questions and half of these improvements were statistically significant. However, as for the change in attitude, although there was an improvement in all domains, only the medication-related management domain showed a statistically significant improvement in score following the training. This could be due to the Type II error caused by the small number of participants, so even if a true statistically significant difference is there, it is not visible. Moreover, change in attitude could only be assessed from the change in practice, which has to be assessed over some time. Not all participants received the ATM training, which needs ongoing support to embed what was learned. However, the significant positive shift in attitude towards medication use allows us to be optimistic that, if fully implemented and practised as intended, SPECTROM would improve staff attitudes toward the person who displays challenging behaviour.

The data gathered through feedback questionnaires and interviews show a positive impact of SPECTROM on staff knowledge and attitude. SPECTROM was perceived as practical, applicable, acceptable, and relevant to staff’s training and is complementary than contradictory to the available training within their own organisations. The handouts, some internal resources such as the accessible medication leaflets, the Yellow Book and the size of the training was found helpful. However, time seems to be a limiting factor in staff benefitting fully from the SPECTROM resources. This was further compounded by the restraint composed by the COVID-19 pandemic. One suggestion is to involve service managers so that they could approve the training and allow support staff time to explore SPECTROM resources in more detail. We, therefore, are planning for our future feasibility study to train several service managers who will then roll out the training among the support staff they manage. In that way, both service managers and the support staff will be fully involved in the training.

### 4.1. Strengths

This is the first training programme developed specifically to address the issue of overprescribing of psychotropic medication in adults with intellectual disabilities, and the field-testing data (both quantitative and qualitative) show a positive impact of SPECTROM on support staff and seem to have achieved its objectives by not only providing the necessary knowledge to staff, but also giving them the confidence to effectively liaise with doctors and other professionals and family carers. The training seems to also have motivated support staff to more often use a psychosocial approach to help adults with intellectual disabilities who are distressed, and to concentrate more on the person they support, by helping them to build skills, rather than the challenging behaviour.

### 4.2. Limitations

Several limitations have made the interpretation of the findings of this study difficult. Because of the COVID-19 pandemic, it was difficult to carry out the field testing as the care homes for adults with intellectual disabilities were affected badly, and as a result, the support staff and the participants of the field testing were under immense pressure. It was, therefore, difficult to recruit participants and deliver the training of both modules to all participants. As a result, the overall number of participants has remained low. Moreover, as we had to adapt two questionnaires from previous studies, some of the questions appeared ambiguous for participants in the field of intellectual disabilities to answer, and, thus, perhaps not providing an accurate score. The psychometric properties of the two adapted questionnaires and the purpose-designed questionnaires we developed to capture both trainees’ and trainers’ feedback were not established among adults with intellectual disabilities; although, the trainee feedback questionnaire items have shown good internal consistency. Although the training was found helpful, we do not know whether this achieved its ultimate goal in reducing the overmedication of adults with intellectual disabilities. Hence, we propose to carry out a larger scale feasibility and implementation study in preparation for a future RCT to assess the cost and clinical effectiveness of SPECTROM in reducing overmedication.

### 4.3. Summary

Quantitative data showed support staff had more knowledge on psychotropic medications post-training, with statically better responses on 8 out of 16 questions. Similarly, for the MAVAS-R-ID scale, although there was a shift towards positive attitude after the training, only the medication management subdomain was significantly different. There was a greater disagreement with the use of medications to manage challenging behaviour after the training (See Figure 2). Furthermore, all trainees and trainers agreed on the applicability, acceptability, practicality, and relevance of SPECTROM, which was measured using a purpose-designed questionnaire. Qualitative data also showed SPECTROM had a positive impact on support staff. Participants found SPECTROM training helped to: (a) change attitude in addressing challenging behaviour, (b) improve self-reflection, (c) improve knowledge, (d) improve staff support provided to PwID, and (e) feel empowered and confident in the management of challenging behaviour.

## 5. Conclusions

SPECTROM training is one of the first training for support staff to help reduce the overmedication of PwID that focussed on improving staff’s understanding of psychotropic medications and the medication review process, promoting a holistic approach at managing challenging behaviour, and understanding PwID and reasons for their challenging behaviour. Results of a small field test show SPECTROM has positive significant impact on support staff’s knowledge on psychotropic medications and attitude towards challenging behaviour and its management. Due to the small number of participants included in this study, a future large RCT is required to identify whether the ultimate goal in reducing overmedication of adults with intellectual disabilities can be achieved through training support staff in SPECTROM.

## Figures and Tables

**Table 1:** Summary domain scores on MAVAS-R-ID scale.

Domains	Total Pooled Median at Baseline (25th–75th Percentile)	Total Pooled Median Post-Training (25th–75th Percentile)	Wilcoxon Signed-Rank Test Results
1: Internal causative factors	10 (8–11.75)	10 (8.25–12)	Z = −0.539, *p* = 0.590
2: External causative factors	12 (10–14.75)	12 (10–13)	Z = −0.932, *p* = 0.351
3: Situational/interactional causative factors	15 (13–18.50)	16(14–18)	Z = −1.056, *p* = 0.291
4: Management-medication	13 (11–15.75)	14.50 (12–16)	Z = −2.039, *p* = 0.041
5: Management-non-medical	12.50 (11–14.50)	12.50 (11–15)	Z = −0.857, *p* = 0.391

**Table 2:** Summary scores according to the trainee questionnaire.

	Number of Items	Cronbach’s Alpha	Mean (SD)	The Proportion of Participants Showing Response above Mean Neutral Score (A Combined Score on ‘Somewhat Agree’ and ‘Agree Completely’)
Total scale	38	0.957	161.75 (18.92)	100% over 114
Applicability	17	0.917	71.92 (8.76)	100% over 51
Acceptability	10	0.888	43.42 (6.05)	100% over 30
Practicality	8	0.942	32.92 (5.57)	100% over 24
Relevance	3	0.730	13.50 (1.51)	100% over 9

**Table 3:** Trainers’ scores on the Likert scale.

Domains	The Proportion of Participants Showing Response above Mean Neutral Score (‘Somewhat Agree’ or ‘Agree Completely’)
Total	100% over 126
Applicability	100% over 36
Acceptability	100% over 60
Practicality	100% over 18
Relevance	100% over 12

## Data Availability

The Principal Investigator will preserve the confidentiality of participants taking part in the study and fulfil transparency requirements under the General Data Protection Regulation for health and care research. Only the designated trial investigators have access to the personal data of the participants and to the final dataset. The focus group transcripts are anonymised, and support staff outcome data are the property of the sponsor (CNWL Partnership NHS Foundation Trust, UK). Regulatory bodies if appropriate may request access to personal data. However, no personal data were collected in this study. There may be opportunity to share data anonymously if necessary and appropriate after proper authorisation and approval are obtained.

## Supplementary Materials

The following are available online at https://www.mdpi.com/article/10.3390/ijerph182413161/s1, Supplementary Material S1: Psychotropic knowledge questionnaire. Supplementary Material S2: MAVAS-R-ID. Supplementary Material S3: Trainee feedback questionnaire. Supplementary Material S4: Trainer feedback questionnaire. Supplementary Material S5: Trainer’s proforma. Supplementary Material S6: Interview topic guide.
