# Supplementary material for: Short-Term Psycho-Education for Caregivers to Reduce Overmedication of People with Intellectual Disabilities (SPECTROM): Development and Field Testing
Source: Int J Environ Res Public Health. 2021 Dec 14;18(24):13161. doi: 10.3390/ijerph182413161 (PMC8701820; doi:10.3390/ijerph182413161)
Supplement: Supplementary file 1 [file ijerph-18-13161-s001.zip › S1 Psychotropic knowledge questionnaire.pdf]

Psychotropic knowledge questionnaire

(Adapted from de Kuijper & van der Putten, Journal of Applied Research in ID, 2017)

Carer ID……………………………………

Date……………………………………......

Baseline, pre-training [ ]

Follow up 1 (post training) [ ]

Follow up 2 (6 months post training) [ ]

Please circle the answer for each item that you think is right.

How many different classes of psychotropic medications are there	1-3	>3	Don't know
The most frequently prescribed classes for treatment of challenging behaviours are	Anti-epileptics	Anti-psychotics	Don't know
How much time does antidepressants usually take to be effective in improving patient's mood?	Within two days	Within 2-4 weeks	Don't know
How much time antipsychotics generally take to show a positive effect on the psychotic symptoms of patients?	0-2 weeks	Over 2 weeks	Don't know
Sleeping pills are a type of psychotropic medication.	Yes	No	Don't know

Methylphenidate is used for the treatment of	ADHD	ASD	Don’t know
Risperidone is an antidepressant drug.	Yes	No	Don’t know
Sertraline is an antidepressant drug.	Yes	No	Don’t know
Some antiepileptic drugs are also used for treating challenging behaviour.	Yes	No	Don’t know
Following are the common side effects of risperidone			
Weight gain.	Yes	No	Don’t know
Drowsiness.	Yes	No	Don’t know
Muscle wasting.	Yes	No	Don’t know
Muscle stiffness.	Yes	No	Don’t know
Shaky hands.	Yes	No	Don’t know
Life threatening skin rash.	Yes	No	Don’t know
Antipsychotic drugs are useful in treating core symptoms of autism.	Yes	No	Don’t know

Professor Shoumi Deb, MBBS, FRCPsych, MD, Imperial College London, UK. Email: s.deb@imperial.ac.uk
