# Supplementary material for: Short-Term Psycho-Education for Caregivers to Reduce Overmedication of People with Intellectual Disabilities (SPECTROM): Development and Field Testing
Source: Int J Environ Res Public Health. 2021 Dec 14;18(24):13161. doi: 10.3390/ijerph182413161 (PMC8701820; doi:10.3390/ijerph182413161)
Supplement: Supplementary file 1 [file ijerph-18-13161-s001.zip › S2 REVISED MAVAS-R ID carer attitude scale.pdf]

# Carer attitude to management of challenging behaviour

(**MAVAS-R-ID**; adapted from Duxbury et al., 2008)

Carer ID: ……………………………Carer age:……………………………………….

Date: ………………………………………………………………………………..

Assessment: Baseline (pre-training) [ ]
Follow-up 1 (post-training) [ ]
Follow-up 2 (6 months) [ ]

When scoring, please keep the following points in mind:

a) Your own honest views and beliefs should be reflected in the answers rather than your organisation's or others' views.
b) ID in this scale stands for intellectual (learning) disability.
c) Answer the questions as per your own experience.
d) Read the questions carefully as some items are written in the reverse order thus making negative of negatives as positive.

For each item, tick the box that reflects your own view most honestly. Ignore the 'Total score' section.

**DO NOT DELIBERATE TOO LONG ON EACH ITEM – YOUR FIRST REACTION IS USUALLY THE RIGHT ONE.**

| Items | Strongly agree<br>1 | Agree<br>2 | Neither agree/ nor disagree<br>3 | Disagree<br>4 | Strongly disagree<br>5 |
|---|---|---|---|---|---|
| **Internal causative factors** | | | | | |
| It is difficult to prevent people with ID from becoming aggressive | | | | | |
| People with ID are aggressive because they are ill | | | | | |
| There are types of people with ID who are aggressive | | | | | |
| **Total sub-score** | | | | | |
| | | | | | |
| **External causative factors** | Strongly disagree<br>1 | Disagree<br>2 | Neither agree/ nor disagree<br>3 | Agree<br>4 | Strongly agree<br>5 |
| People with ID are aggressive because of the environment they are in | | | | | |

| | | | | | |
|---|---|---|---|---|---|
| Restrictive environments can contribute towards aggression | | | | | |
| If the physical environment were different, persons with ID would be less aggressive | | | | | |
| **Total sub-score** | | | | | |
| | | | | | |
| **Situational/ interactional causative factors** | Strongly disagree 1 | Disagree 2 | Neither agree/ nor disagree 3 | Agree 4 | Strongly agree 5 |
| Other people may make people with ID aggressive or violent | | | | | |
| Poor communication between the person with ID and the carers may lead to aggression in the person with ID | | | | | |
| Improved one-to-one relationships between carers and the person with ID can reduce the incidence of aggression | | | | | |
| It is largely situations that contribute towards the expression of aggression by the person with ID | | | | | |
| **Total sub-score** | | | | | |
| | | | | | |
| **Management-medication** | Strongly agree 1 | Agree 2 | Neither agree/ nor disagree 3 | Disagree 4 | Strongly disagree 5 |
| Medication is a valuable approach for treating aggression and violent behaviour in a person with ID | | | | | |
| Prescribed medication should be used more frequently for aggressive behaviour in persons with ID | | | | | |
| There is strong scientific evidence that medication reduces aggression in all people with ID | | | | | |
| There are a large number of medications currently licenced to manage aggressive behaviour in people with ID | | | | | |
| **Total sub-score** | | | | | |
| | | | | | |
| **Management-non-medical** | Strongly disagree 1 | Disagree 2 | Neither agree nor disagree 3 | Agree 4 | Strongly agree 5 |

| | | | | | |
|---|---|---|---|---|---|
| Negotiation/ better communication could be used more effectively when managing aggression in a person with ID | | | | | |
| Alternatives to the use of restrictive practices and medications could be used more frequently to manage aggression in a person with ID | | | | | |
| The use of de-escalation is successful in preventing aggression and violence in persons with ID | | | | | |
| **Total sub score** | | | | | |
| | | | | | |
| **Grand Total score** | | | | | |

Professor Shoumi Deb, MBBS, FRCPsych, MD, Imperial College London, UK.
Email: s.deb@imperial.ac.uk
