# Supplementary material for: Short-Term Psycho-Education for Caregivers to Reduce Overmedication of People with Intellectual Disabilities (SPECTROM): Development and Field Testing
Source: Int J Environ Res Public Health. 2021 Dec 14;18(24):13161. doi: 10.3390/ijerph182413161 (PMC8701820; doi:10.3390/ijerph182413161)
Supplement: Supplementary file 1 [file ijerph-18-13161-s001.zip › s3 Trainee likert scale.pdf]

SPECTROM

Trainee questionnaire

Initials of the trainee………………………………………………….

Date completed……………………………………….

Please rate the following items from 'Disagree completely' to 'Agree completely.'

Applicability

Please rate the following statements.	1 Disagree completely	2 Disagree somewhat	3 Do not agree or disagree	4 Agree somewhat	5 Agree completely
I find the training useful in my day to day practice.					
I will use/check the information available in SPECTROM while discussing care planning of people that I support.					
I will make use of the checklists/monitoring sheets for antipsychotics available in the modules.					
I will make use of the resources developed by SPECTROM.					
The training helped me to understand psychotropic medications and their use in people with intellectual (learning) disabilities.					
The training helped me to understand better when to use and when not to use psychotropic medication.					
The training helped me to understand the side effects of psychotropic medication better.					
The training helped me to understand the reasons for challenging behaviour better.					
The training helped me to understand the person better who show challenging behaviour.					
The training helped me to understand the medication review and medication withdrawal process better.					
CATS will help me to assess triggers for challenging behaviour better in the person I support.					

Yellow book/passport is useful in storing information regarding person's medications and preferences.					
Accessible psychotropic medication leaflets are useful in explaining medications to people I support.					
External resources are useful for gathering important information that could be used in day to day practice.					
The training has helped me to change my practice for better.					
The training has changed my attitude to challenging behaviour.					
The training has changed my attitude to the person showing the behaviour.					
Total:					

Acceptability

Please rate the following statements	1 Disagree Completely	2 Disagree somewhat	3 Do not agree or disagree	4 Agree somewhat	5 Agree completely
The training material is easy to understand.					
Contents of the training are at the right level for my knowledge and expertise.					
The training itself is not exhausting.					
The pace of delivery of training is right.					
The length of time needed to complete the training is right.					
The SPECTROM site is easy to navigate through.					
Case studies are helpful.					
Group discussions are helpful.					
Video clips are useful.					
Tasks are useful.					
MCQs are useful.					
Total:					

Practicality

Please rate the following statements	1 Disagree Completely	2 Disagree somewhat	3 Do not agree or disagree	4 Agree somewhat	5 Agree completely
The training will help me to address problems during care planning of people I support.					
The training will help me to liaise better with relevant professionals involved in the care of the person I support.					
The training will help me to liaise better with family carers of the person I support.					
The training will help me to engage better with the person I support.					
The training will help me to concentrate on the skills building of the person I support rather than concentrate on her/his behaviour.					
The training will give me confidence to ask the doctors right questions.					
The training will help me to take the right information to the doctor's medication review.					
The training will give me confidence in carrying out team medication review on a regular basis.					
Total:					

Relevance

Please rate the following statements	1 Disagree Completely	2 Disagree somewhat	3 Do not agree or disagree	4 Agree somewhat	5 Agree completely
The training is relevant to my personal development.					
SPECTROM provides useful training which complements other training I have received.					
The training will help eventually to reduce overmedication of people with intellectual disabilities.					
Total:					

Free text

Please write any other comments below in the box (particularly what you think is missing from the training and also SPECTROM modules, how could the training and the SPECTROM site be made any better etc.).
