# Supplementary material for: Short-Term Psycho-Education for Caregivers to Reduce Overmedication of People with Intellectual Disabilities (SPECTROM): Development and Field Testing
Source: Int J Environ Res Public Health. 2021 Dec 14;18(24):13161. doi: 10.3390/ijerph182413161 (PMC8701820; doi:10.3390/ijerph182413161)
Supplement: Supplementary file 1 [file ijerph-18-13161-s001.zip › S4 Trainer likert scale.pdf]

Trainer questionnaire

Initials of the trainer……………………………………………………

Date completed…………………………………………

Please rate the following items from ‘Disagree completely’ to ‘Agree completely.’

Applicability

Please rate the following statements	1 Disagree Completely	2 Disagree somewhat	3 Do not agree or disagree	4 Agree somewhat	5 Agree completely
The training will be useful for staff’s day to day practice.					
The training will help support staff understand the side effects of psychotropic medication better.					
The training will give support staff confidence to ask doctors the right questions.					
The training will help support staff understand the reasons for challenging behaviour better.					
The training will help support staff understand the person they support better.					
The training will help change support staff’s practice for better.					
CATS is useful to assess triggers of behaviours.					
Accessible psychotropic medication leaflets are useful in explaining medications to people with intellectual disabilities.					
The yellow book is helpful in storing valuable information about the person with intellectual disabilities.					
The training will change support staff’s attitude to challenging behaviour.					
The training will change support staff’s attitude to the person showing the behaviour.					
External resources are useful for gathering important information that could be used in support staff’s day to day practice.					

Acceptability

Please rate the following statements	1 Disagree Completely	2 Disagree somewhat	3 Do not agree or disagree	4 Agree somewhat	5 Agree completely
It is easy to prepare for the SPECTROM training.					
I had time to read the manual of the training.					
The manual's instructions are easy to understand.					
The content of the core module is easy to understand.					
The length of information on core module is right.					
The questions and instructions in each core module are clear.					
It is easy to locate the manuals, handouts and core modules on the website.					
The website is easy to use.					
I had time to read other modules in addition to core modules and corresponding manual.					
It took more time to complete the training than I anticipated.					
I completed the whole training in one day.					
I had enough time to go through all the contents and tasks.					
It was easy to deliver the training.					
The handouts were helpful.					
The video clips were engaging.					
The group discussions were helpful.					
The tasks were appropriate for the training.					
The case studies were helpful and appropriate.					
The pace of delivery of training was right.					
At the end of the training, I asked trainees to explore SPECTROM webpage further as part of their homework					

Practicality

Please rate the following statements	1 Disagree Completely	2 Disagree somewhat	3 Do not agree or disagree	4 Agree somewhat	5 Agree completely

The information in SPECTROM will help support staff gain confidence in carrying out team medication review on a regular basis.					
The information in SPECTROM can be used as reference points when discussing care planning of people with intellectual disabilities.					
The training will help support staff engage better with the person they support.					
The training will help support staff concentrate on the skills building of the person they support rather than concentrate on her/his behaviour.					
The training will help support staff liaise better with relevant professionals involved in the care of the person they support.					
The training will help support staff liaise better with family carers of the person they support.					

Relevance

Please rate the following statements	1 Disagree Completely	2 Disagree somewhat	3 Do not agree or disagree	4 Agree somewhat	5 Agree completely
I recommend the training to others.					
The training complements other trainings in the service.					
The training is relevant to support staff's personal development.					
The training will help eventually to reduce overmedication of people with intellectual disabilities.					

Free text

Please write any other comments below in the box (particularly what you think is missing from the training and also SPECTROM modules, how could the training and the SPECTROM site be made any better etc.).
