# Supplementary material for: Short-Term Psycho-Education for Caregivers to Reduce Overmedication of People with Intellectual Disabilities (SPECTROM): Development and Field Testing
Source: Int J Environ Res Public Health. 2021 Dec 14;18(24):13161. doi: 10.3390/ijerph182413161 (PMC8701820; doi:10.3390/ijerph182413161)
Supplement: Supplementary file 1 [file ijerph-18-13161-s001.zip › S5 Trainer proforma.pdf]

Trainer's Proforma

SPECTROM

Please read the questions carefully and answer each question.

Trainer's initials	
Date of training	
Number of participants on the day of the training	
What was the mix of trainee's background like (e.g. senior vs. new staff, manager vs. staff)?	

Which core module did you deliver?

____

Before the training

How much time did it take you to prepare for the training?	
How much time did it take to read the whole manual of your chosen topic (including the Introduction manual) to prepare for the training?	
What did you think about the manual?	
What did you think about the contents of the core module?	
What did you think about the format of the core module (e.g. group discussion, video clips, case studies, handouts)?	
What did you think about the layout and contents of the core modules on the SPECTROM website?	
Did you have time to read other modules in addition to core modules and corresponding manual? If so, how many modules did you read and what were they?	

What did you think about the rest of the modules on the SPECTROM site (e.g. are they useful, is there too much information or too little, ease of navigating through the site and move from one module to another or to internal and external resources?	
Do you know whether any of the trainees explored SPECTROM webpage after training?	

During training

How much time did it take to deliver the training? When did you start and finish and how many breaks did you take?	
Was it easy to find participants for the training?	
Did you use all the handouts provided?	
What did you think about the handouts?	
What did the trainees think of the handouts?	
What did you think about the tasks?	
What did the trainees think about the tasks?	
What did you think about the case studies?	
What did trainees think about the case studies?	
What did you think of the video clips?	
What did the trainees think about the video clips?	
Did you manage to engage the trainees adequately in the discussion?	
If you had to skip any task or content, please write which tasks or contents you skipped?	
Were the contents of the module useful to the trainees or are they below or above their level of knowledge/expectation?	

What was the speed/pace of the training? Was the training well-paced or did you think you had to rush the training?	
For those who delivered Alternative to Medication core module, what did you think of the meditation at the end of training? Did you think this was helpful?<br><br>Please skip this question if you delivered the medication core module.	
What challenges did you face during the training?<br><br>Please mention any barriers you had in preparing and delivering the training.	

After the training

Have you or any trainee used CATS?	
What did you think of CATS?	
What did trainees think about CATS?	
What did you think of accessible medication leaflets?	
What did the trainees think about the accessible medication leaflets?	
What did you think about the Yellow book?	
What did the trainees think about the Yellow book?	
What did you think about the external hyperlinked resources?	
What did the trainees think about the external hyperlinked resources?	

What did you like most about the training?

What did you like least about the training? If you could change anything, what would you change?

Please write any other comments below.
