# Supplementary material for: Short-Term Psycho-Education for Caregivers to Reduce Overmedication of People with Intellectual Disabilities (SPECTROM): Development and Field Testing
Source: Int J Environ Res Public Health. 2021 Dec 14;18(24):13161. doi: 10.3390/ijerph182413161 (PMC8701820; doi:10.3390/ijerph182413161)
Supplement: Supplementary file 1 [file ijerph-18-13161-s001.zip › s6 topic guide.pdf]

What is your general impression of the training?

What did you find particularly helpful?

What did you find not so useful?

What would you like to change in the training if any?

Were the contents below or above your level of knowledge?

What did you think about the overall time taken for the training (pace of delivery)?

What did you think of the format of the training?

What did you think of the case studies?

What did you think of the discussion and your involvement in that?

What did you think of the video clips?

What did you think of the handouts?

What did you think of the tasks?

What did you think of the homework?

Did you have a chance to explore the SPECTROM site after the training session?

What did you think of the site (easy to navigate through, important information accessed through hyperlinks, too much information, tool little information, too confusing, hyperlinks are useful so that you can take your time to explore each module in detail etc.)?

What did you think of the MCQs?

What did you think of CATS (would this be useful in your day to day practice, would you use it)?

What did you think of the Yellow book?

What did you think of the accessible medication leaflets (will you use them, when and how)?

Would you be able to use information in SPECTROM on a regular basis (if so, how)?

Has your own practice changed anyway because of the training (how)?

Has your attitude to addressing challenging behaviour or use of psychotropic medication for that changed because of the training (how)?

Do you think this training will be useful to your colleagues (why, how and if not, why not)?
